# Species- and strain-specific differences in the phagocytosis of *Prototheca*: insights from live-cell imaging

**DOI:** 10.1128/iai.00066-23

**Published:** 2023-08-18

**Authors:** Mohammed J. A. Haider, Christopher D. Shave, Chinaemerem U. Onyishi, Tomasz Jagielski, Samuel Lara-Reyna, Eva-Maria Frickel, Robin C. May

**Affiliations:** 1 Institute of Microbiology and Infection, School of Biosciences, College of Life and Environmental Sciences, University of Birmingham, Birmingham, United Kingdom; 2 Department of Medical Microbiology, Institute of Microbiology, Faculty of Biology, University of Warsaw, I. Miecznikowa, Warszawa, Poland; Yale University School of Medicine, New Haven, Connecticut, USA

**Keywords:** *Prototheca*, phagocytosis, macrophages, confocal microscopy, imaging, pathogenesis

## Abstract

The genus *Prototheca* is an extremely unusual group of achlorophyllic, obligately heterotrophic algae. Six species have been identified as pathogens of vertebrates, including cattle and humans. In cattle, *P. bovis* is the main infectious pathogen and is associated with bovine mastitis. In contrast, human infections typically involve *P. wickerhamii* and are associated with a spectrum of varying clinical presentations. *Prototheca* spp. enter the host from the environment and are therefore likely to be initially recognized by cells of the innate immune system. However, little is known about the nature of the interaction between *Prototheca* spp. and host phagocytes. In the present study, we adopt a live-cell imaging approach to investigate these interactions over time. Using environmental and clinical strains, we show that *P. bovis* cells are readily internalized and processed by macrophages, whereas these immune cells struggle to internalize *P. wickerhamii*. Serum opsonization of *P. wickerhamii* only marginally improves phagocytosis, suggesting that this species (but not *P. bovis*) may have evolved mechanisms to evade phagocytosis. Furthermore, we show that inhibition of the kinases Syk or PI3K, which are both critical for innate immune signaling, drastically reduces the uptake of *P. bovis*. Finally, we show that genetic ablation of MyD88, a signaling adaptor critical for Toll-like receptor signaling, has little impact on uptake but significantly prolongs phagosome maturation once *P. bovis* is internalized. Together, our data suggest that these two pathogenic *Prototheca* spp. have very different host-pathogen interactions which have potential therapeutic implications for the treatment of human and animal disease.

## INTRODUCTION

*Prototheca* constitutes a genus of highly unusual eukaryotes. These organisms are nonphotosynthetic, obligately heterotrophic algae and are closely related to the genus *Chlorella* (1). To date, six members have been identified as pathogens of vertebrates. Of these, two are particularly important: *Prototheca bovis* (formerly, *Prototheca zopfii* genotype 2) (2) and *Prototheca wickerhamii*. The former is the main causative agent of infection in cattle, whereas the latter is the major cause of human disease. In cattle, infection predominantly manifests as either acute or chronic mastitis; in both cases, infection lowers milk yield and drastically impacts animal welfare (3 - 9). The route of entry is thought to be environmental, via the teat orifice, or via contaminated milking equipment (4). In humans, infection in immunocompetent individuals may be localized (e.g., olecranon bursitis) or cutaneous, and is thought to be a result of traumatic inoculation of the pathogen via wounds. Infections in immunocompromised individuals, particularly those with cell-mediated immunity defects, may either be localized or systemic (10 - 14).

There is still a paucity of information on the relevant virulence factors used by *Prototheca* spp. A few studies on *P. bovis* and *Prototheca ciferrii* have highlighted a role for survival and replication in the phagolysosome as well as induction of mitochondrial apoptosis (15) through nuclear factor kappa light chain enhancer of activated B-cells (NF-κB)- and NLRP3-dependent pathways (16). Biofilm formation appears to enhance *Prototheca* species-associated pathology and resistance to various sanitizers (17, 18). Additionally, biofilm formation enhances immune evasion as peripheral blood mononuclear cells (PBMCs) were shown to produce an early proinflammatory cytokine (interleukin (IL)-6) in response to planktonic, but not biofilm-associated, *P. wickerhamii* (19).

Previously, the murine macrophage cell line J774A.1 has been shown to internalize *P. bovis* up to 8 h post-infection (15). This internalization correlated with increased mRNA expression of proapoptotic factors, including cytochrome *c*, caspase-3, caspase-9, and *Bax*. To date, however, no temporal investigation of this host-pathogen interaction has been undertaken, and consequently, little is known about the pattern recognition receptors (PRRs) and downstream pathways involved in *Prototheca* pathogenesis.

In this study, we used a live-cell imaging approach to dissect the spatiotemporal aspects of phagocytosis in finer detail. We focused on *P. bovis* and *P. wickerhamii* since they are the major causative agents of infection in cattle and humans, respectively. Our *in vitro* model showed that most uptake events for *P. bovis* occur rapidly (i.e., within 5–10 min of infection). Intriguingly, *P. wickerhamii* was not phagocytosed at all, either by J774A.1 cells or by immortalized bone marrow-derived macrophages (iBMDMs). Serum opsonization of *P. wickerhamii* only marginally enhanced phagocytic uptake compared with *P. bovis*. Interestingly, human monocyte-derived macrophages (MDMs) were marginally better than murine cells at engulfing unopsonized *P. wickerhamii* but still exhibited much poorer phagocytosis of this species relative to *P. bovis*. Furthermore, we showed that pharmacological inhibition of critical signaling kinases correlated with drastically impaired uptake of *P. bovis*. Finally, using iBMDMs deficient in MyD88, a signaling adaptor critically required for almost all Toll-like receptors (TLRs), we were able to show that genetic ablation of this protein drastically prolongs the time required for phagosome maturation. Altogether, our data provide novel insight into the dynamic nature of *P. bovis* and *P. wickerhamii* phagocytosis by macrophages.

## RESULTS

### Unopsonized *P. wickerhamii* cells are not phagocytosed by J774A.1 cells

**FIG 1:**
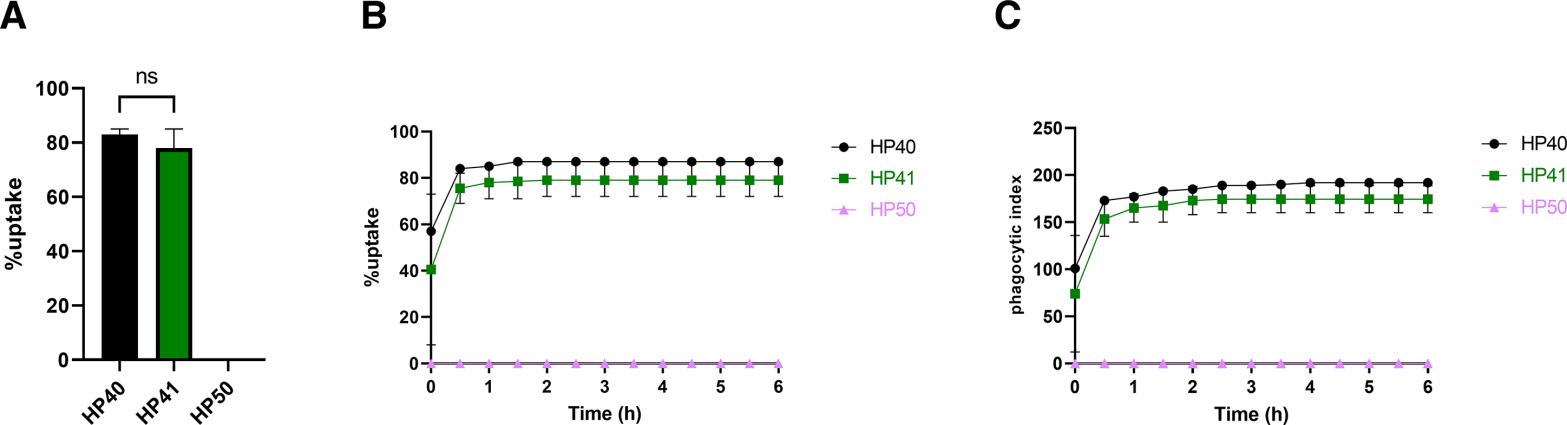
Unopsonized *P. wickerhamii* (HP50) cells are not phagocytosed by J774A.1 cells. (**A**) J774A.1 cells were seeded at 1 × 10^5^/well in complete medium and incubated at 37°C and 5% CO_2_. The next day, cells were challenged with HP40 (*Prototheca bovis*, clinical strain), HP41 (*Prototheca bovis*, environmental strain), or HP50 (*Prototheca wickerhamii*, clinical strain) at an MOI = 3 and imaged every 3 min for 1 h on a Zeiss Axio Observer live microscope using a ×20 objective. Data show the percentage of uptake and are the mean ± SEM of two independent experiments. Statistical significance was assessed by an unpaired *t*-test where *p* < 0.05 was considered significant. (**B**) Co-cultures were set up as described in panel** A**, but imaging continued for 6 h at the same rate. Data show the percentage of uptake and are the mean ± SEM of two independent experiments. (**C**) Data show the phagocytic index. Percentage of uptake is defined as the percentage of macrophages that takes up at least one algal cell at any point in time. Phagocytic index is defined as the total number of algal cells taken up by at least 100 macrophages at any point in time. SEM, standard error of the mean.

To better understand the time-dependent differences in uptake of *Prototheca* spp., we exposed phorbol 12-myristate 13-acetate (PMA)-activated J774A.1 cells to a clinical and an environmental isolate of *P. bovis* (HP40 and HP41, respectively) or a clinical isolate of *P. wickerhamii* (HP50) at an MOI = 3 (alga:macrophage ratio) in serum-free medium for 1 h. By the end of the imaging period, approximately 80% of analyzed macrophages had successfully internalized HP40 or HP41 (Fig. 1A; Video S1 and S2), but remarkably, we saw no instances of uptake for *P. wickerhamii* (Fig. 1A). To see if HP50 uptake occurs at a delayed timepoint, we extended the imaging period up to 6 h, but even at this extended time, we saw no evidence of phagocytosis (Fig. 1B and C). In contrast, uptake of HP40 and HP41 was robust. To test whether this result with *P. wickerhamii* is strain specific, we tested an additional environmental strain (HP52, isolated from sewage filter sand in the USA). However, two independent experiments, imaging over 300 macrophages in total, revealed only one instance of phagocytosis (data not shown). Thus, it appears that unopsonized *P. wickerhamii* is essentially not phagocytosed by murine macrophages.

To dissect these uptake events in greater detail, we analyzed phagosome closure time (defined as the time from initial point of contact between macrophage and algal cell until complete internalization), lysosomal fusion (defined as the time from phagosome closure until formation of a red halo using the dye LysoTracker Red [LTR], indicative of early phagosome maturation), and variations in the numbers of cells internalized by actively phagocytosing macrophages. Approximately 40% and 50% of HP40 and HP41 phagosomes were completely internalized within 10 min of contact with macrophages, respectively (Fig. S1A). Of these, around 45% of HP40 phagosomes matured within 5 min of phagosome closure, and around 45% of HP41 phagosomes matured within 10 min of phagosome closure (Fig. S1B). Most phagocytosing macrophages internalized a single *Prototheca* cell, although a significant proportion internalized two or more (Fig. S1C).

To quantify phagocytosis independently of imaging, we assessed viability of *Prototheca* spp. post-exposure to J774A.1 cells in co-cultures (Fig. S1D). *P. bovis* HP40 and HP41 showed approximately 20% and 21% recovery, whereas *P. wickerhamii* HP50 showed around 75% recovery with respect to the initial inoculum density. These data are in accordance with those observed in Fig. 1, suggesting that macrophages are effective at engulfing and killing *P. bovis*, but not *P. wickerhamii*.

### Human MDMs phagocytose *P. wickerhamii* less readily than *P. bovis*

*P. wickerhamii* is the main causative agent of human protothecosis. Because J774A.1 cells are of murine origin, we tested whether a similar pattern would occur with human phagocytes. We exposed strains of *P. bovis* (HP3 and HP40) and *P. wickerhamii* (HP50 and HP52) to human monocyte-derived macrophages and performed endpoint analysis after imaging for 1 h (Video S3 and S4).

**FIG 2:**
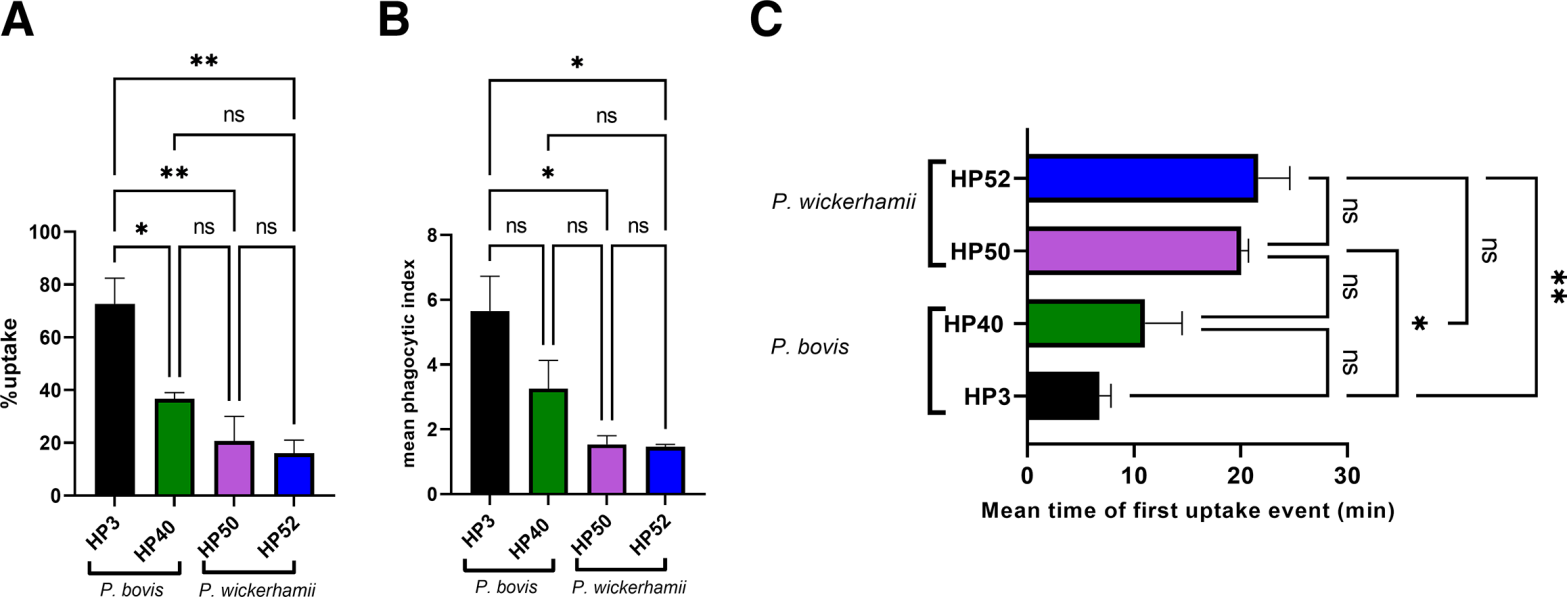
Human MDMs phagocytose *P. wickerhamii* less readily compared with *P. bovis*. MDMs were cultured as described in Materials and Methods with an initial monocyte seeding density of 5 × 10^5^/well in complete medium and incubation at 37°C and 5% CO_2_. On the day of the experiment, the medium was replaced with serum-free medium containing different strains of *P. bovis* (HP3 and HP40) or *P. wickerhamii* (HP50 and HP52) at an MOI = 1. Co-cultures were then imaged every minute for 1 h on a Nikon Eclipse Ti live microscope using a ×20 objective. Data show the mean ± SEM of three independent experiments. At least 50 macrophages were analyzed per condition, per experiment. (**A**) Percentage of uptake is defined as the percentage of macrophages that have phagocytosed at least one algal cell by the end of the imaging period. (**B**) Mean phagocytic index is defined as the mean number of algal cells taken up by actively phagocytosing macrophages by the end of the imaging period. (**C**) Mean time of first uptake event is defined as the mean time (in minutes) required for an actively phagocytosing macrophage to take up an algal cell for the first time. Statistical significance was assessed by one-way analysis of variance followed by Tukey’s multiple comparisons test, where *p* < 0.05 was considered significant. *p < 0.05, **p < 0.01. ns, not significant.

The percentage of uptake of *P. bovis* strains ranged between approximately 38% and 75%, which was significantly higher than that observed for *P. wickerhamii* strains (<20% uptake) (Fig. 2A). Furthermore, human MDMs internalized more *P. bovis* cells overall (Fig. 2B) and engulfed them much earlier during the imaging period compared with *P. wickerhamii* (Fig. 2C). Consequently, although human MDMs are capable of phagocytosing *P. wickerhamii* (unlike murine cells), they retain a far higher capacity to engulf *P. bovis*.

### Wild-type (WT) iBMDMs phagocytose HP40 and HP41 in a similar manner to J774A.1 cells

**FIG 3:**
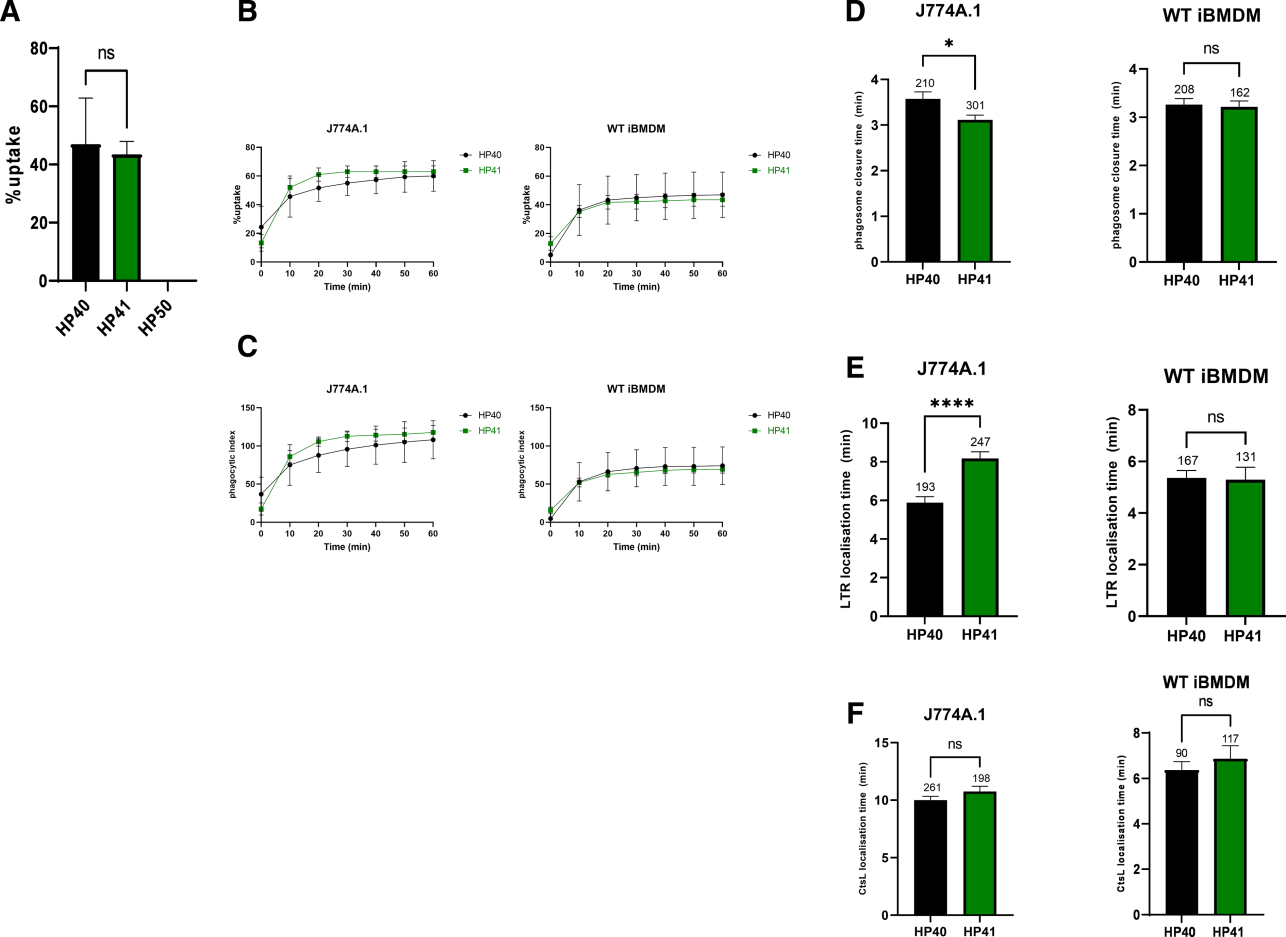
J774A.1 cells and WT iBMDMs show similar phagocytosis dynamics for the internalization of *P. bovis*. (**A**) WT iBMDMs were seeded at 5 × 10^4^/well and incubated at 37°C and 5% CO_2_. The next day, cells were challenged with unopsonized HP50 at an MOI = 3 and imaged every 30 s for 1 h on a Nikon Eclipse Ti live microscope using a ×20 objective. Data show the percentage of uptake and are expressed as the mean ± SEM of three independent experiments. (B through F) J774A.1 cells and WT iBMDMs were cultured as described for panel **A** and challenged with HP40 or HP41 at an MOI = 3 for 1 h in the presence of LTR or CtsL. Cells were imaged every 30 s on a Nikon Eclipse Ti live microscope using a ×20 objective. Data show (**B**) percentage of uptake, (**C**) phagocytic index, (**D**) phagosome closure time, (**E**) LTR localization time, and (**F**) CtsL localization time. Data are the mean ± SEM of three independent experiments. For panels D through F, the numbers above each bar represent the total number of phagosomes analyzed. For panels **A **and D through F, statistical significance was assessed by an unpaired *t*-test where *p* < 0.05 was considered significant. *p < 0.05, ****p < 0.0001. For panels B and C, statistical significance was assessed by two-way analysis of variance followed by Šídák’s multiple comparisons test where *p* < 0.05 was considered significant. Percentage of uptake is defined as the percentage of macrophages that take up at least one algal cell at any point in time. Phagocytic index is defined as the total number of algal cells taken up by at least 100 macrophages at any point in time. LTR localization time is defined as the time from the completion of phagosome closure until the formation of a red LTR halo. CtsL localization time is defined as the time from the completion of phagosome closure until the formation of a red CtsL halo. CtsL, cathepsin L.

To further test whether our observations in murine cells may be cell line specific, we analyzed uptake of *Prototheca* cells by WT immortalized bone marrow-derived macrophages. Macrophages were exposed to HP40, HP41, or HP50 at an MOI = 3 and imaged for 1 h. Intriguingly, as noted in Fig. 1A, WT iBMDMs similarly failed to internalize HP50, whereas the rate of uptake was around 50% and 45% for HP40 and HP41, respectively (Fig. 3A). Thus, the failure of *P. wickerhamii* to be phagocytosed is not unique to J774A.1 cells but likely represents a generic evasion of macrophage recognition.

We then imaged HP40 and HP41 internalization by WT iBMDMs at higher temporal resolution (Fig. 3; Video S5 and S6) in order to compare phagosome closure and maturation between the two cell lines. Percentage of uptake (Fig. 3B) and phagocytic index (Fig. 3C) did not vary greatly between HP40 and HP41 although interestingly were consistently lower than uptake by J774A.1 macrophages. In J774A.1 cells, there was a modest but statistically significant difference in the mean phagosome closure time observed for HP41 vs HP40 (3 min vs 3.5 min, respectively) (Fig. 3D). In contrast, LTR localization was somewhat slower for HP41 vs HP40 (Fig. 3E).

We then assessed late phagosome maturation by monitoring the localization of Magic Red cathepsin L (CtsL), a dye that becomes activated by the action of cathepsin L, a late phagosomal protease (Video S7 to S10). We observed no difference in the time required for either HP40 or HP41 phagosomes to localize with CtsL in both J774A.1 cells and WT iBMDMs, although in general, CtsL localization occurred more rapidly in WT iBMDMs compared with J774A.1 cells (around 6.0–6.5 min or 10–11 min post-internalization, respectively) (Fig. 3F). Thus, it appears that, although speed of uptake and early phagosome maturation vary slightly between these two *Prototheca* strains, ultimately phagosomal maturation completes at a similar time for both.

### Opsonization of HP50 with adult human serum enhances phagocytic uptake by WT iBMDMs

**FIG 4:**
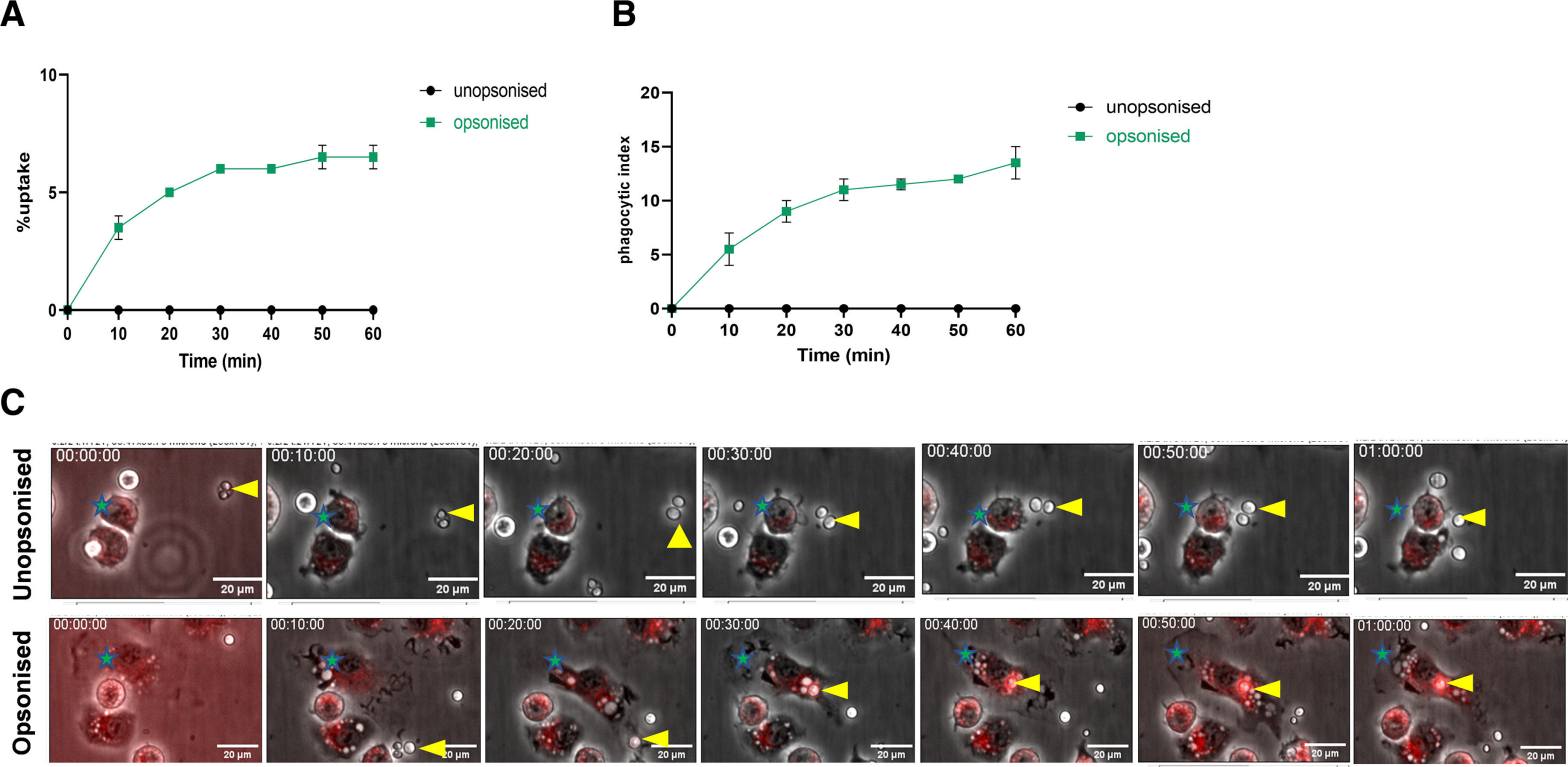
Opsonization of *P. wickerhamii* (HP50) results in marginal enhancement of uptake. (**A**) WT iBMDMs were seeded at 5 × 10^4^/well and incubated at 37°C and 5% CO_2_. The next day, cells were challenged with unopsonized or human serum-opsonized HP50 at an MOI = 3 in the presence of LTR. Cells were then imaged every 30 s for 1 h on a Nikon Eclipse Ti live microscope using a ×20 objective. Data show the percentage of uptake and are expressed as the mean ± SEM of two independent experiments. (**B**) Phagocytic index. (**C**) Representative still images of unopsonized (top) vs opsonized (bottom) HP50 phagocytosis by WT iBMDMs stained with LTR. Green stars represent macrophages. Yellow arrowheads represent HP50 cells. Still images are shown at 10-min increments up to the end of the imaging period (1 h). Scale bar = 20 µm. Note: the y-axis in panels **A **and **B **has been adjusted differently from that used in Fig. 1 and 3 to emphasize the difference in uptake and phagocytic index between unopsonized and opsonized HP50, given the very small number of cells taken up. Percentage of uptake is defined as the percentage of macrophages that take up at least one algal cell at any point in time. Phagocytic index is defined as the total number of algal cells taken up by at least 100 macrophages at any point in time.

Having observed no phagocytic uptake of unopsonized HP50 in both J774A.1 cells and WT iBMDMs, we wondered whether opsonizing the pathogen with adult serum may facilitate enhanced uptake. Since *Prototheca* spp. are widespread, it is likely that most adults have opsonizing antibodies to this organism, as well as complement opsonins. We therefore exposed WT iBMDMs to HP50 cells that had been exposed to adult human serum for 1 h and analyzed the uptake events (Video S11). We observed a gradual increase in both percentage of uptake (Fig. 4A) and phagocytic index (Fig. 4B) throughout the imaging period. Remarkably, however, even under these conditions, the uptake of *P. wickerhamii* remained extremely low, with only 6% of macrophages having phagocytosed at least one algal cell by the end of the imaging period (Fig. 4C). For the few opsonized HP50 cells that were internalized, we analyzed phagosome closure time and LTR localization time. These were approximately 3.3 and 6.2 min, respectively (Tables S12). Together, these data suggest that serum opsonization of HP50 facilitates phagocytosis by WT iBMDMs but only marginally.

### Pharmacological inhibition of critical kinases associated with signaling and actin reorganization drastically reduces uptake of HP40 and HP41

There is currently a paucity of knowledge on the exact recognition mechanisms involved in the uptake of *Prototheca* spp. by host phagocytes. Furthermore, it is not clear which classes of pattern recognition receptors are involved, if at all. To begin addressing these issues, we exposed WT iBMDMs to two pharmacological inhibitors: piceatannol and wortmannin. Piceatannol inhibits Syk, a cytosolic tyrosine kinase that binds to the phosphorylated immunoreceptor tyrosine-based activation motifs of various receptors involved in innate immunity, including the phagocytic Fc receptors and C-type lectin receptors (CLRs) (20). Wortmannin inhibits phosphoinositide 3-kinase (PI3K), a lipid-modifying enzyme central to a large array of cellular metabolic pathways and immune mechanisms, including actin cytoskeletal remodeling and membrane closure during phagocytosis (21 - 23).

**FIG 5:**
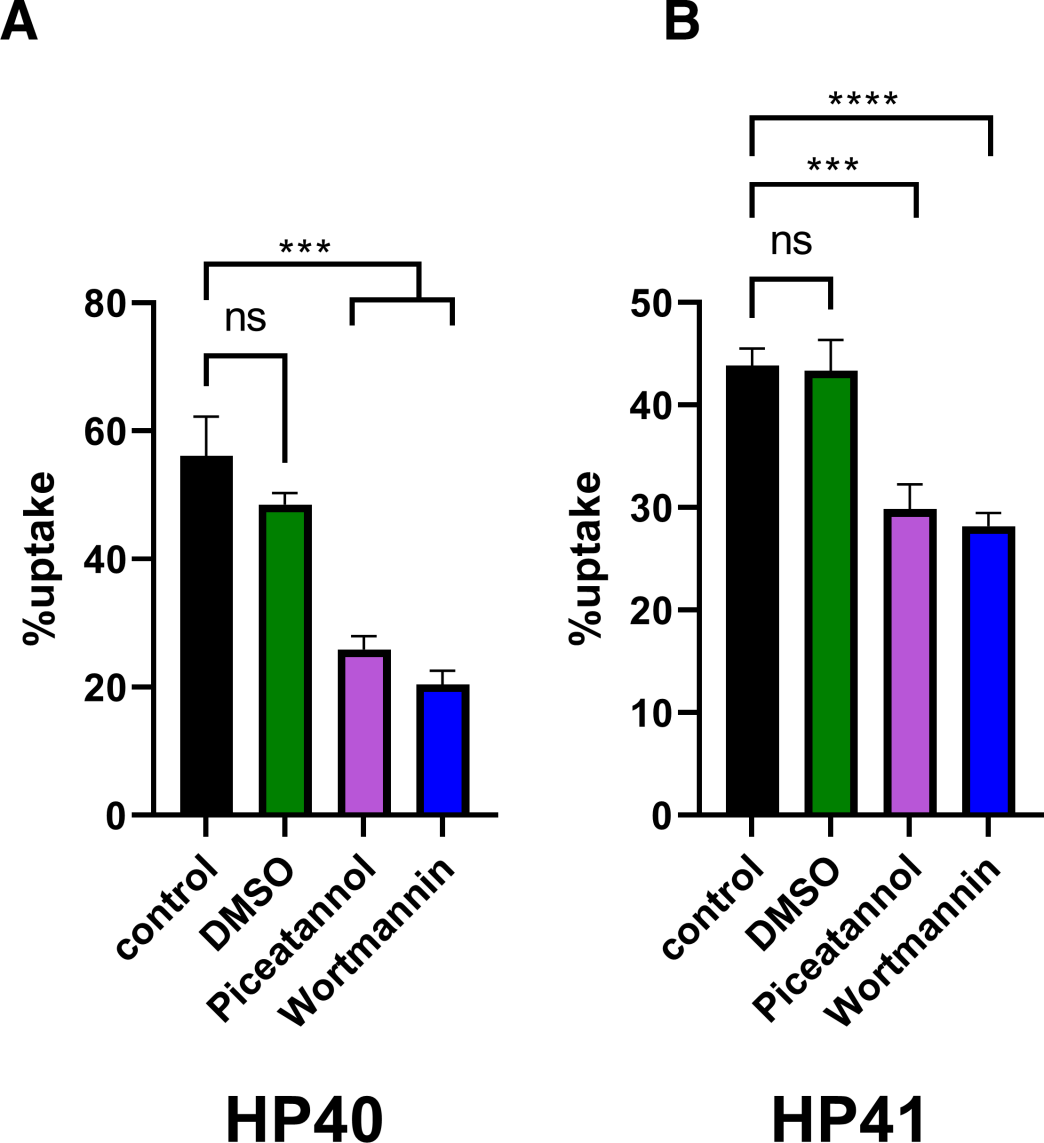
Pharmacological inhibition of Syk or PI3K significantly impacts the uptake of *P. bovis* (HP40 and HP41). WT iBMDMs were seeded at 5 × 10^4^/well and incubated at 37°C and 5% CO_2_. The next day, cells were treated for 1 h with dimethyl sulfoxide (DMSO) (1 mM), the Syk inhibitor Piceatannol at 75 µM, or the PI3K inhibitor Wortmannin at 20 µM and then challenged for 1 h with HP40 or HP41 at an MOI = 3. Cells were then fixed in 4% paraformaldehyde, washed, and imaged on a Nikon Eclipse Ti Live microscope using a ×20 objective. Data show the percentage of uptake for (**A**) HP40 and (**B**) HP41 and are expressed as the mean ± SEM of three independent experiments. Statistical significance was assessed by an unpaired *t*-test where *p* < 0.05 was considered significant. ***p < 0.001, ****p < 0.0001. Percentage of uptake is defined as the percentage of macrophages that take up at least one algal cell at any point in time.

Inhibition of WT iBMDMs with either piceatannol or wortmannin significantly reduced uptake of both HP40 (Fig. 5A) and HP41 (Fig. 5B). The two drugs had minor effects on early phagosome maturation (Fig. S3A and B) but no impact on later maturation (Fig. S3C and D). Thus, these data suggest that inhibition of Syk or PI3K drastically impacts uptake and internalization but has little impact on phagosome maturation once internalization is complete.

### Genetic ablation of MyD88 has no impact on uptake but prolongs the time required for early phagosome maturation

Because PI3K is known to be activated downstream of various PRRs, including TLRs, we next wondered whether genetic ablation of MyD88 would compromise the dynamics of *P. bovis* phagocytosis. MyD88 is a signaling adaptor critical for the function of all known TLRs, except TLR3, which uses TRIF. Thus, complete loss of MyD88 would be expected to compromise TLR-dependent physiological outcomes. To address this, we challenged MyD88^−/−^ or TRIF^−/−^ iBMDMs with HP40 or HP41 as described previously and imaged co-cultures for 1 h.

**Fig 6:**
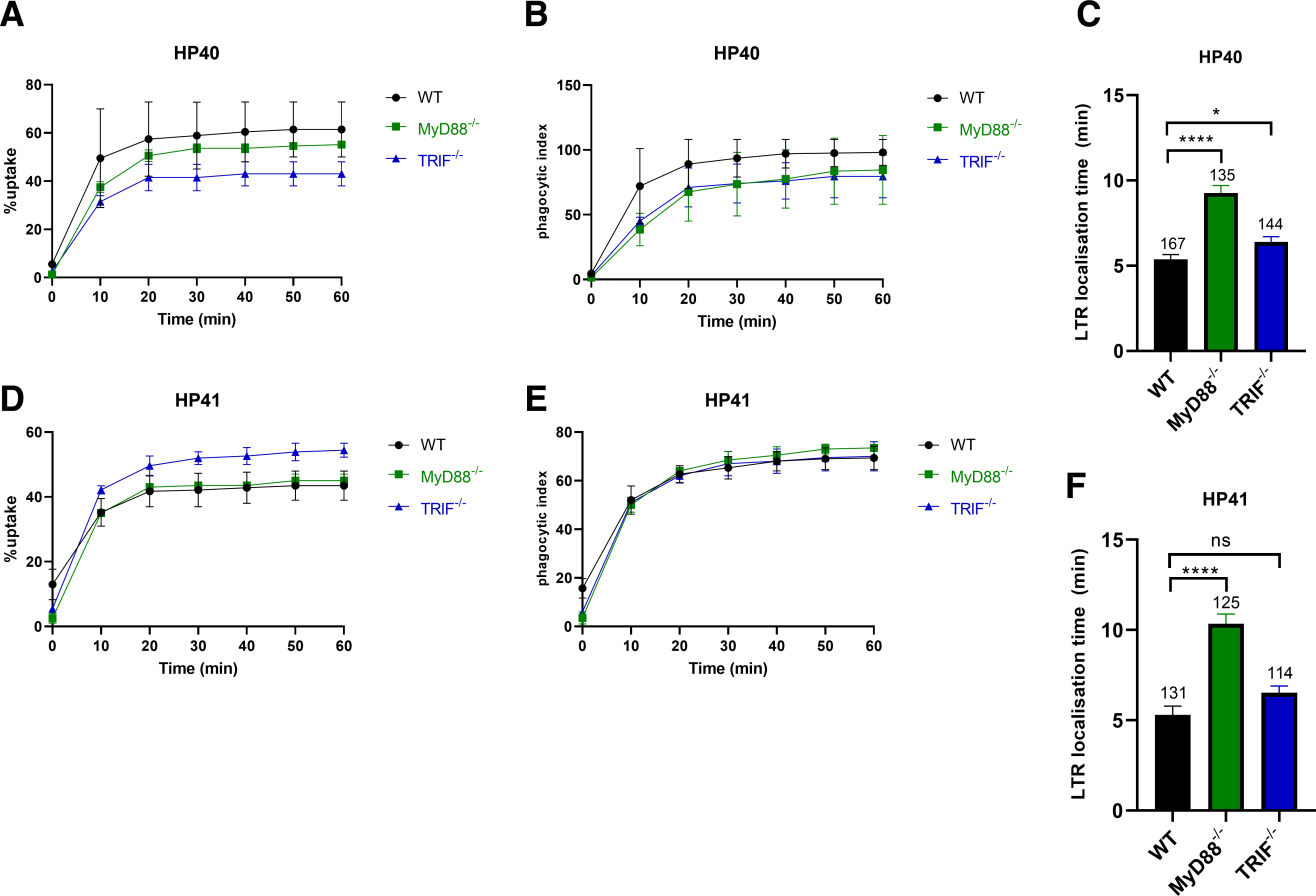
*P. bovis* phagosome maturation is significantly impacted in MyD88^−/−^ iBMDMs. WT, MyD88^−/−^, and TRIF^−/−^ iBMDMs were seeded at 5 × 10^4^/well and incubated at 37°C and 5% CO_2_. The next day, cells were challenged with HP40 or HP41 at an MOI = 3 in the presence of LTR and imaged every 30 s for 1 h on a Nikon Eclipse Ti live microscope using a ×20 objective. Data are expressed as the mean ± SEM of at least two independent experiments. (**A and D**) Percentage of uptake. (**B and E**) Phagocytic index. (**C and F**) LTR localization time. For panels** C **and **F**, the numbers above each bar represent the total number of phagosomes analyzed. For panels A, B, D, and E, statistical significance was assessed by two-way analysis of variance followed by Dunnett’s multiple comparisons test, where *p <* 0.05 was considered significant. For panels** C and F**, statistical significance was assessed by an unpaired *t*-test where *p* < 0.05 was considered significant. *p < 0.05, ****p < 0.0001. Percentage of uptake is defined as the percentage of macrophages that take up at least one algal cell at any point in time. Phagocytic index is defined as the total number of algal cells taken up by at least 100 macrophages at any point in time. LTR localization time is defined as the time from the completion of phagosome closure until the formation of a red LTR halo.

Neither HP40 nor HP41 showed a significant change in uptake in knockout iBMDMs, but, once internalized, early phagosome maturation was notably delayed for both isolates. This was especially the case for MyD88^−/−^ iBMDMs, whereas TRIF^−/−^ iBMDMs showed a minor, but significant, delay in phagosome maturation for HP40 but not HP41 (Fig. 6C and F). Furthermore, analysis of LTR localization time in finer increments showed a significant decrease in the percentage of HP40 and HP41 phagosomes that had localized with LTR within 5 min of phagosome closure in MyD88^−/−^ iBMDMs compared with WT iBMDMs (around 4.0-fold and 6.9-fold decrease, respectively) (Fig. S4A and B). Together, these data suggest that TLR signaling critically impacts the early maturation, but not uptake, of phagosomes containing HP40 or HP41 in iBMDMs.

## DISCUSSION

Protothecosis is a rare algal infection with varying clinical presentations in humans and nonhuman vertebrates (24). In 2017, Todd et al. reported that only 211 cases of human protothecosis had been published worldwide up to April 2017 (25), although these numbers appear to be rising (26, 27). Of more significance is *Prototheca*-associated bovine mastitis, a subtype of mammary gland inflammation with drastic impact on the dairy industry worldwide (6, 28, 29). Presently, no specific treatment is available for *Prototheca*-associated bovine mastitis; however, infection control strategies generally include culling infected cows and/or drying all infected quarters. Importantly, the source and route of transmission are still unknown, although it is postulated that contaminated milking equipment is a plausible route. An earlier Japanese study evaluated fecal samples from herds with/without a history of protothecal mastitis; the study identified *P. zopfii* genotype 2 (now redefined as *P. bovis*) in all fecal samples obtained from calves from herds with a history of protothecal mastitis but not from those without a history of protothecal mastitis. The authors therefore suggested that this may be a case of persistent intestinal infection, implicating feces as a likely source of *P. bovis* (5).

Few studies have been published on the innate immune response to *Prototheca* spp. In one study, it was shown that *in vitro* challenge of bovine mammary epithelial cells (bMECs) with *P. zopfii* genotypes 1 and 2 (now *P. ciferrii* and *P. bovis*, respectively) correlated with elevated expression of inflammatory markers at the gene and protein levels (30). mRNA expression of four PRRs, including TLR2, TLR4, NOD1, and NOD2, peaked between 3 and 6 h post-stimulation, suggesting the involvement of both cell-surface and cytosolic PRRs in the recognition and response to this pathogen. Specifically, this was most evident for *P. bovis*, therein implying its increased immunogenicity compared with *P. ciferrii*. Likewise, challenge with *P. bovis* correlated with increased mRNA expression of IL-1β, IL-8, and tumor necrosis factor (TNF), all of which are critical acute-phase proinflammatory cytokines. Importantly, this was paralleled by increased nuclear translocation of NF-κB, a transcription factor central to inflammation.

In a later study by the same group, it was shown that challenge with *P. bovis* correlated with mitochondrial apoptosis (15). Analysis of total RNA from bMECs, murine mammary epithelial cells, and J774A.1 cells challenged with *P. bovis* showed an increase in the expression of TNF, IL-8/Cxcl-1, Bax, Apaf-1, cytochrome *c*, caspase-3, and caspase-9. This was paralleled by a decrease in the expression of Bcl-2, an antiapoptotic factor, as well as an increase in mitochondrial membrane depolarization. The authors therefore suggest that *P. bovis* triggers mitochondrial membrane damage, which releases cytochrome *c* and initiates apoptasome formation and a signaling cascade culminating in apoptotic cell death.

A more recent publication by the same group corroborated these earlier findings and expanded on the nature of mitochondrial apoptosis triggered by *P. bovis* infection (16). Using serological, biochemical, and microscopic approaches, the authors showed increased mitochondrial membrane damage and reactive oxygen species (ROS) accumulation in bMECs challenged with either *P. ciferrii* or *P. bovis*, with a more drastic effect observed for *P. bovis*. In line with their previous observations (15), the authors identified an NF-κB-dependent induction of proinflammatory cytokine production. Furthermore, components of the NLRP3 inflammasome activation pathway, including NLRP3 itself, caspase-1, caspase-1 p20, and ASC, were all elevated at the protein level 12 h post-infection. This correlated with an increased expression level and secretion of both IL-1β and IL-18, two proinflammatory cytokines central to inflammasome signaling and activation. Importantly, these observations were abrogated upon treatment of bMECs with (2-(2,2,6,6-Tetramethylpiperidin-1-oxyl-4-ylamino)-2-oxoethyl)triphenylphosphonium chloride (mito-TEMPO), a ROS scavenger. Hence, the authors suggest that infection of bMECs with *P. ciferrii*, but more importantly *P. bovis*, triggers an NF-κB-dependent overexpression of proinflammatory cytokines through generation of mitochondrial ROS.

Our current study focused on dissecting the temporal dynamics of *P. bovis* and *P. wickerhamii* phagocytosis by J774A.1 cells and murine iBMDMs. To the best of our knowledge, this is the first study to evaluate in detail the time-dependent changes in uptake, phagosome closure, and phagosome maturation for the selected isolates. We showed that *P. bovis* was readily engulfed, and the overall dynamics of uptake and phagosome maturation did not differ greatly between environmental and clinical (mastitis-associated) strains. Furthermore, we showed that unopsonized *P. wickerhamii* is not phagocytosed, either by J774A.1 or WT iBMDMs. Even opsonization with adult human serum only resulted in a marginal increase in the phagocytosis of *P. wickerhamii*. Thus, this species appears not to be phagocytosed by macrophages of murine origin.

We showed that unopsonized *P. wickerhamii* can be phagocytosed by human MDMs, although (as with murine phagocytes) uptake of *P. bovis* is far more efficient. At present, there is no information on the precise PRRs required for recognition and/or internalization of *Prototheca* spp. Our data suggest that *P. bovis* may be phagocytosed using one set of receptors that are expressed on both human and murine macrophages, whereas *P. wickerhamii* may require an independent set of receptors expressed only on human macrophages (and perhaps at low levels, reflecting the poor uptake rate). To date, there has been very little work to identify the specific surface components of *Prototheca* that may be recognized by phagocyte receptors. Sporopollenin is a potential component that may be recognized and may facilitate internalization (31), although the receptor for this molecule remains unknown. Earlier studies have identified glucose-, galactose-, mannose-, and hexosamine-based structures in cell walls of *Prototheca* spp. (32), but these do not appear to bear any structural resemblance to chitin or cellulose found in fungal and plant cells, respectively. Furthermore, β-glucan has been reported in *P. bovis* (formerly *P. zopfii* genotype 2) (33), but this is β-(1,4)-glucan rather than β-(1,3) and β-(1,6) structures commonly found in fungal cell walls.

Earlier studies have shown phagocytosis by macrophages of either single cells or clusters of sporangia containing numerous sporangiospores (34 - 39), primarily based on histological findings from human, canine, feline, or goat samples. Our study primarily focused on phagocytes of murine origin (iBMDMs and J774A.1 cells), neither of which appear to engulf *P. wickerhamii*. Thus, it may be that murine macrophages do not express a critical receptor required for *P. wickerhamii* (but *P. bovis* or *P. ciferrii*) uptake. Alternatively, perhaps the histological findings reflect a particular *in vivo* behavior of macrophages that is not recapitulated by *in vitro* conditions. Either way, in the future, more work will be needed to characterize species-dependent differences in the phagocytic abilities of murine vs nonmurine macrophages.

Pharmacological inhibition of WT iBMDMs with wortmannin, a PI3K inhibitor, resulted in a significantly reduced uptake of both HP40 and HP41. Among others, PI3K has important downstream effects on actin remodeling necessary for phagocytosis. Our findings thus suggest internalization of *P. bovis* is a “classical” phagocytosis event involving Syk-dependent receptors and PI3K activation. This contrasts with earlier findings suggesting that internalization of *P. bovis* is an active, microbe-driven process rather than a phagocyte-specific response (15). This discrepancy between our observations and those of the earlier report may be due to the specific identify of the *P. bovis* strain used, the culture medium, and/or the multiplicity of infection chosen for the assay.

That MyD88^−/−^ and TRIF^−/−^ iBMDMs internalize *P. bovis* in the same manner as WT iBMDMs but show defects in phagosome maturation hints at the importance of TLR signaling for the phagocytic processing of this pathogen. Moreover, these findings are supported by earlier observations of elevated mRNA expression of TLRs upon challenging bMECs with *P. ciferrii* or *P. bovis*, in particular, TLR2 and TLR4 (30). The specific identify of the TLRs involved in recognition, binding, and uptake of *P. bovis* remains to be determined. Thus, it appears that host phagocyte interactions with *P. bovis* depend on collaborative, perhaps redundant, recognition by multiple classes of membrane-associated or cytosolic PRRs, including TLRs, CLRs, and NLRs. This is not altogether surprising in that other eukaryotic pathogens have previously been shown to be recognized, bound, and/or internalized by multiple receptors (40, 41).

In conclusion, we show that *P. wickerhamii*, the main causative agent of human protothecosis, is not internalized efficiently by murine macrophages and is only weakly engulfed by human phagocytes. Further, we show that an environmental and clinical isolate of *P. bovis* is internalized and processed in largely the same way by murine macrophages. Additionally, we show that perturbation of two critical kinases, Syk and PI3K, negatively impacts uptake of *P. bovis*, hence implicating the importance of tyrosine kinase-associated signaling cascades and actin/membrane remodeling in the uptake and processing of this pathogen. Lastly, we show that genetic ablation of MyD88, a signaling adaptor critical to the function of all TLRs except TLR3, correlates with delayed phagosome maturation, but not uptake, of phagosomes containing *P. bovis*. Future work should address the underlying mechanisms involved in species- and strain-specific differences in the uptake and processing of *Prototheca* spp.

## MATERIALS AND METHODS

### Algal cell culture

*Prototheca* strains (apart from HP3) were a kind gift from Dr. Tomasz Jagielski (University of Warsaw) and were described within reference (2). *P. bovis* strain HP3 (strain SAG2021) is a type strain originally isolated from a case of acute bovine mastitis in Germany (kind gift from Prof. Uwe Rösler, Freie Universität Berlin). *P. bovis* strain HP40 (strain E9) was originally isolated from a clinical case of bovine mastitis in Poland. *P. bovis* strain HP41 was originally isolated from cow’s feeder in Poland (SR4). *P. wickerhamii* strain HP50 (UTEX1437, ATCC16523, and NRRL Y-2464) was originally isolated from a clinical case of bovine mastitis in the USA (42). *P. wickerhamii* strain HP52 (JCM 9645, CBS 608.66) was originally isolated from sewage filter sand in the USA. All organisms were grown on *Prototheca* isolation medium agar as described previously but without the addition of 5-FC (43). The solution was autoclaved at 121°C for 15–20 min, allowed to cool, and then poured into petri dishes as required.

For experiments, single colonies were inoculated in 2-mL Sabouraud dextrose broth (Sigma) and incubated at 25°C in a rotatory shaker set at 20 rpm for 48–72 h. Cells were pelleted by centrifugation at 2,700 *× g* for 1 min and then washed 3× in phosphate buffered saline (PBS) at 2,700 *× g* for 1 min/wash. Cells were then counted on a CytoSMART automated cell counter (CytoSMART Technologies, Corning, NJ, USA) and adjusted as required for different experiments.

In some experiments, HP50 was opsonized by incubation in pooled adult human serum (Sigma) for 1 h at 25°C on a rotary shaker set to 20 rpm.

### Murine cell culture

J774A.1 cells were purchased from the European Collection of Authenticated Cell Cultures. Immortalized bone marrow-derived macrophages were originally isolated from wild type, MyD88^−/−^, or TRIF^−/−^ C57Bl/6 mice and were provided as a kind gift from Prof. Clare Bryant, University of Cambridge, Cambridge, UK (44, 45). iBMDMs were routinely cultured in Dulbecco’s Modified Eagle’s Medium (low glucose) (Sigma), supplemented with 10% heat-inactivated fetal bovine serum (FBS) (Sigma), 2-mM L-glutamine (Sigma), and 100-U/ml penicillin-streptomycin (Sigma), and incubated at 37°C and 5% CO_2_ in a humidified incubator. J774A.1 cells were cultured similarly, except non heat-inactivated FBS was used.

### Isolation of human monocytes and differentiation into MDMs

Leukocyte cones, from healthy donors, were obtained from the National Health Service Blood Transfusion and promptly processed within 4 h. Peripheral blood mononuclear cells were isolated from the cones using a standard density gradient centrifugation method. Following a Dulbecco's phosphate buffered saline (DPBS) wash, the blood was mixed with an equal volume of DPBS (containing 2% FBS) and carefully layered onto Lymphoprep (StemCell, Vancouver, Canada). Centrifugation was carried out at 1,100 × *g* for 20 min without applying brakes. The resulting white buffy layer was collected and further washed with DPBS by centrifuging at 300*× g* for 10 min. Red blood cells (RBCs) were removed by using an RBC lysis solution (BioLegend, San Diego, CA, USA) for 10 min at room temperature. CD14^+^ monocytes were then isolated from the PBMCs using immunomagnetic positive selection (Miltenyi Biotec, North Rhine-Westphalia, Germany).

The isolated human monocytes were seeded at a density of 5 × 10^5^ cells per well in tissue culture-treated 24-well plates and were cultured in complete RPMI 1640 medium supplemented with 10% heat-inactivated human serum and 20-ng/mL human granulocyte macrophage colony-stimulating factor(GM-CSF) (PeproTech, London, UK) for macrophage differentiation. Cells were incubated for 6 days, with medium replacement on day 3.

### Cell stimulation and phagocytosis assays

J774A.1 cells and iBMDMs were seeded at 1 × 10^5^/well or 5 × 10^4^/well in 24-well plates (Greiner Bio-One, Gloucestershire, England, UK) and incubated at 37°C and 5% CO_2_. The next day, the medium was removed and replaced with fresh medium containing 150 ng/mL PMA (Sigma), and cells were activated for 1 h at 37°C and 5% CO_2_. The medium was removed and replaced with carbon dioxide-independent medium (Thermo Fisher Scientific, Loughborough, England, UK) supplemented with 10% heat-inactivated FBS and containing *P. bovis* or *P. wickerhamii* at an MOI = 3 (i.e., three algal cells: one macrophage) and 100 nM LysoTracker Red-DND 99 (LTR) (Thermo Fisher Scientific) or Magic Red cathepsin L as per the manufacturer’s instructions. Co-cultures were then imaged for 1 h as described below.

In some experiments, cells were treated for 1 h at 37°C with 1 mM dimethyl sulfoxide (DMSO) as a vehicle control (Sigma), 75-µM piceatannol (Sigma) or 20-µM wortmannin (Selleckchem, Planegg, Germany). Medium was then removed and replaced with fresh medium containing *Prototheca* spp. and cells were co-cultured for 1 h. Medium was then removed and replaced with 4% paraformaldehyde fixative solution (Thermo Fisher Scientific) for 10 min at room temperature. Fixative solution was then gently removed. Cells were washed 3× gently with PBS and then imaged on a Nikon Eclipse Ti Live microscope using a ×20 objective.

Following the 6-day MDM differentiation process described above, the medium was replaced with fresh medium containing 10% heat-inactivated human serum but no GM-CSF. Human MDMs were co-cultured with *P. bovis* or *P. wickerhamii* strains at an MOI = 1 with 5% CO_2_ maintained in the imaging chamber. Cultures were imaged every minute for 1 h.

### Live-cell imaging

Co-cultures were set up as described above and then imaged on a Nikon Eclipse Ti Live microscope using a ×20 objective. Videos were acquired using the NIS Elements Advanced Research Imaging Software v.4.20. Images were captured every 30 s in a humidified chamber set at 37°C. In some experiments, co-cultures were imaged for 6 h on a Zeiss Axio Observer Live microscope using a ×20 objective. For these experiments, images were captured every 3 min in a humidified chamber set at 37°C. In other experiments, images were captured every minute in a humidified chamber set at 37°C and 5% CO_2_.

### Analysis of phagocytosis assays

Time-lapse videos acquired on either the Nikon Eclipse Ti Live microscope or Zeiss Axio Observe Live microscope were exported and analyzed in Fiji (ImageJ). At least 100 macrophages were analyzed per condition, per experiment for the following parameters:

- Percentage of uptake: defined as the percentage of macrophages that take up at least one algal cell at any point in time.
- Phagocytic index: defined as the total number of algal cells taken up by at least 100 macrophages at any point in time.
- Mean phagocytic index: defined as the mean number of algal cells taken up by actively phagocytosing macrophages by the end of the imaging period.
- Mean time of first uptake: defined as the mean time (in minutes) required for an actively phagocytosing macrophage to take up an algal cell for the first time from the initiation of imaging.
- Phagosome closure time: defined as the time from initial point of contact between a macrophage and an algal cell until its complete internalization.
- LTR localization time: defined as the time from the completion of phagosome closure until the formation of a red LTR halo.
- CtsL localization time: defined as the time from the completion of phagosome closure until the formation of a red CtsL halo.

### Statistical analysis

Statistical analysis was done in GraphPad Prism v.9.3.1. At least two independent experiments were performed for each condition. For each experiment, at least 100 macrophages were analyzed. Data are expressed as the mean ± standard error of the mean of at least two independent experiments. Statistical significance was assessed by one-way analysis of variance (ANOVA) followed by Tukey’s multiple comparisons test, two-way ANOVA followed by Šídák’s or Dunnett’s multiple comparisons test, or an unpaired *t*-test where appropriate. In all experiments, *p* < 0.05 was considered significant.

## SUPPLEMENTAL MATERIAL

Fig. S1 iai.00066-23-s0001.tif Uptake and phagosome maturation dynamics for *P. bovis* and *P. wickerhamii* phagosomes.

Fig. S2 iai.00066-23-s0002.tif Uptake and phagosome maturation dynamics for *P. bovis* phagocytosed by J774A.1 cells and WT iBMDMs.

Fig. S3 iai.00066-23-s0003.tif Pharmacological inhibition of Syk or PI3K has variable effects on the maturation of *P. bovis* (HP40 and HP41) phagosomes.

Fig. S4 iai.00066-23-s0004.tif Genetic ablation of MyD88 significantly impacts maturation of *P. bovis* (HP40 or HP41) phagosomes.

Supplemental material legends and descriptions iai.00066-23-s0005.docx Text-based description of supplemental figures, tables, and videos with legends.

Tables S1 and S2 iai.00066-23-s0006.docx *P. wickerhamii* (HP50) phagosome closure time and LTR localization time.

Video S1 iai.00066-23-s0007.mp4 J774A.1 cells stained with LTR phagocytosing *P. bovis* (HP40).

Video S2 iai.00066-23-s0008.avi J774A.1 cells stained with LTR phagocytosing *P. bovis* (HP41).

Video S3 iai.00066-23-s0009.avi Human MDMs stained with LTR phagocytosing *P. bovis* (HP40).

Video S4 iai.00066-23-s0010.avi Human MDMs stained with LTR phagocytosing *P. wickerhamii* (HP50).

Video S5 iai.00066-23-s0011.avi WT iBMDMs stained with LTR phagocytosing *P. bovis* (HP40).

Video S6 iai.00066-23-s0012.mp4 WT iBMDMs stained with LTR phagocytosing *P. bovis* (HP41).

Video S7 iai.00066-23-s0013.avi J774A.1 cells stained with CtsL phagocytosing *P. bovis* (HP40).

Video S8 iai.00066-23-s0014.mp4 J774A.1 cells stained with CtsL phagocytosing *P. bovis* (HP41).

Video S9 iai.00066-23-s0015.avi WT iBMDMs stained with CtsL phagocytosing *P. bovis* (HP40).

Video S10 iai.00066-23-s0016.mp4 WT iBMDMs stained with CtsL phagocytosing *P. bovis* (HP41).

ASM does not own the copyrights to Supplemental Material that may be linked to, or accessed through, an article. The authors have granted ASM a non-exclusive, world-wide license to publish the Supplemental Material files. Please contact the corresponding author directly for reuse.
