# Supplementary figures and images for: Species- and strain-specific differences in the phagocytosis of Prototheca: insights from live-cell imaging
Source: Infect Immun. 2023 Aug 18;91(9):e00066-23. doi: 10.1128/iai.00066-23 (PMC10501220; doi:10.1128/iai.00066-23)

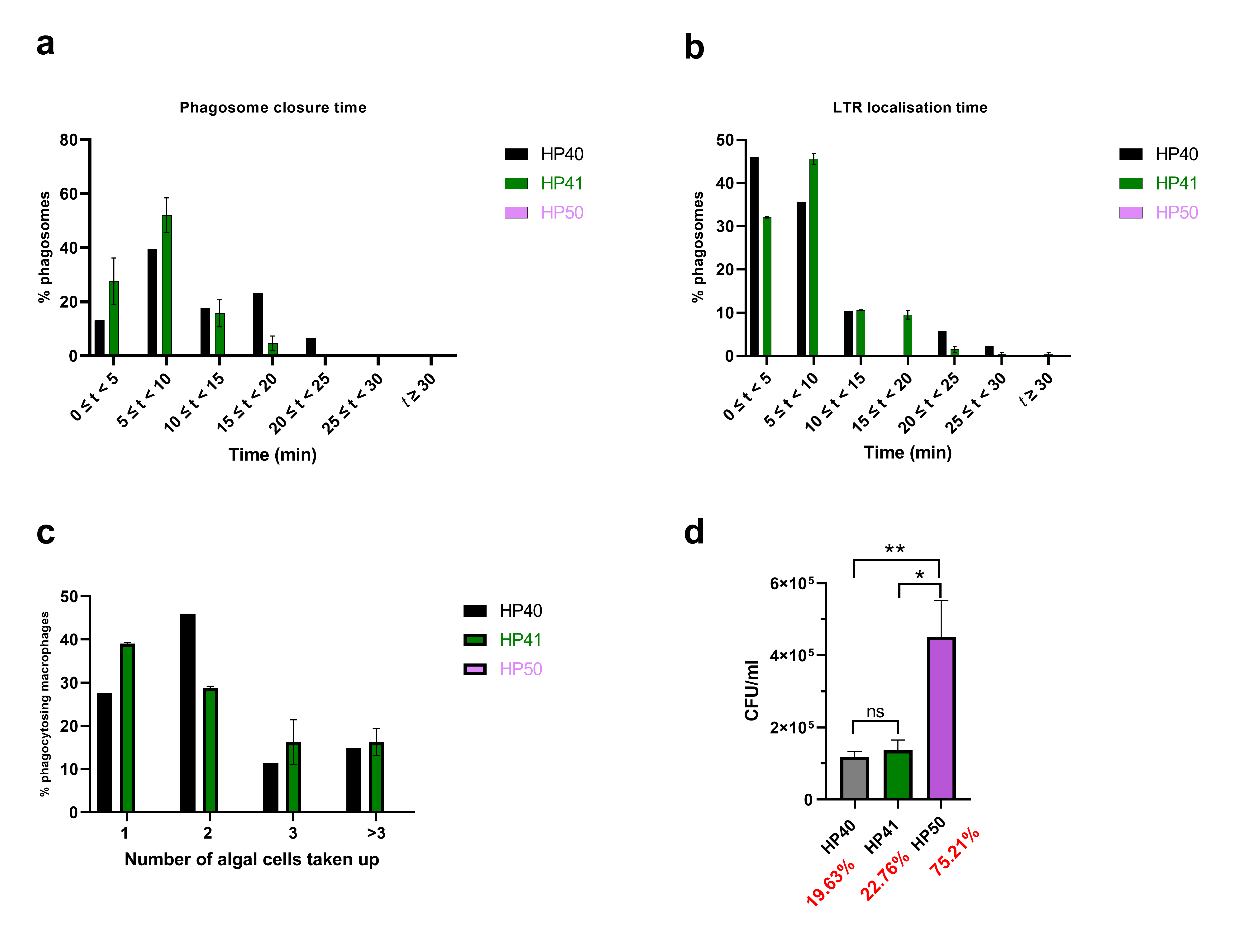

Supplement: Fig. S1 — Uptake and phagosome maturation dynamics for P. bovis and P. wickerhamii phagosomes. [file iai.00066-23-s0001.tif]

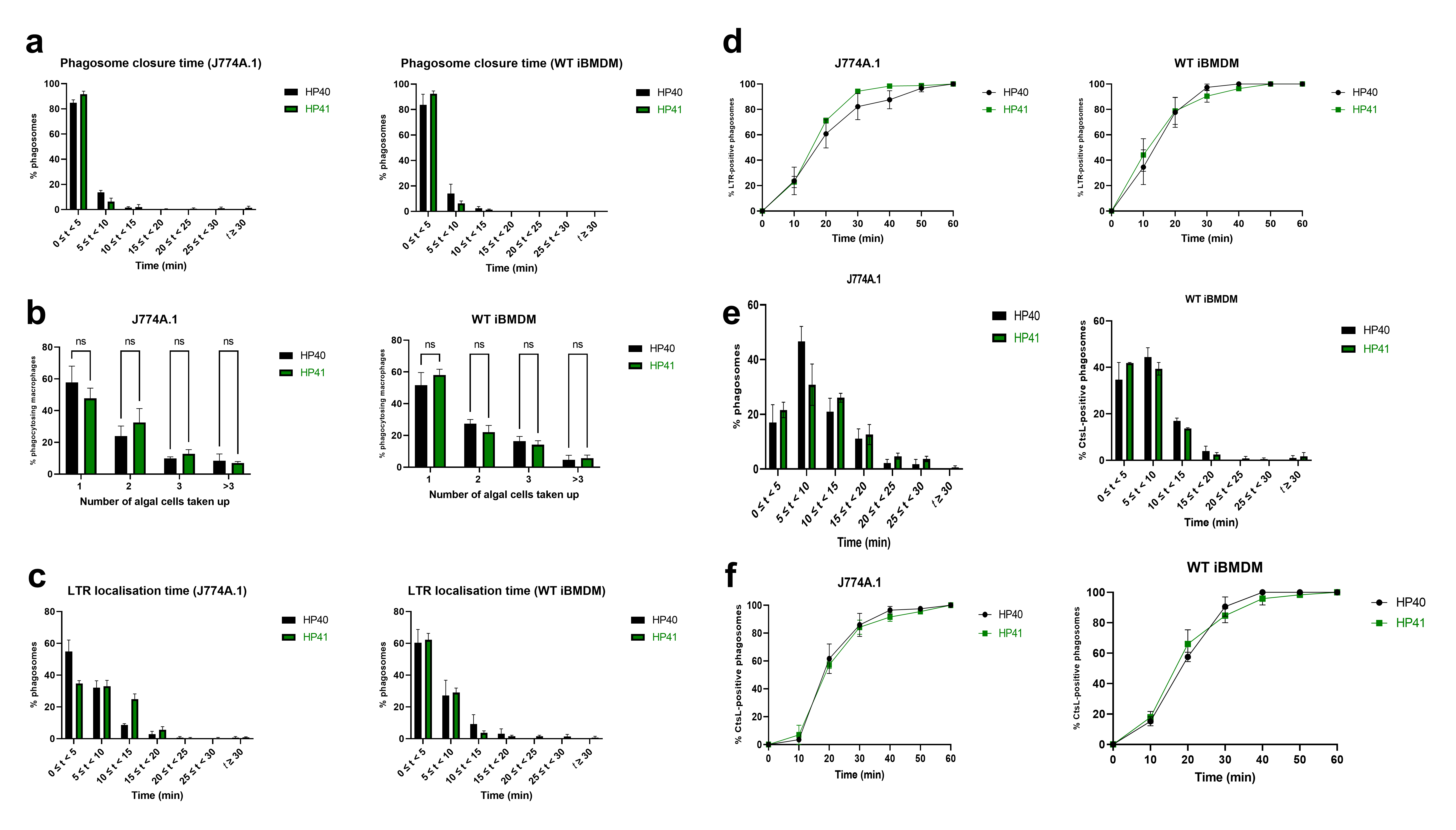

Supplement: Fig. S2 — Uptake and phagosome maturation dynamics for P. bovis phagocytosed by J774A.1 cells and WT iBMDMs. [file iai.00066-23-s0002.tif]

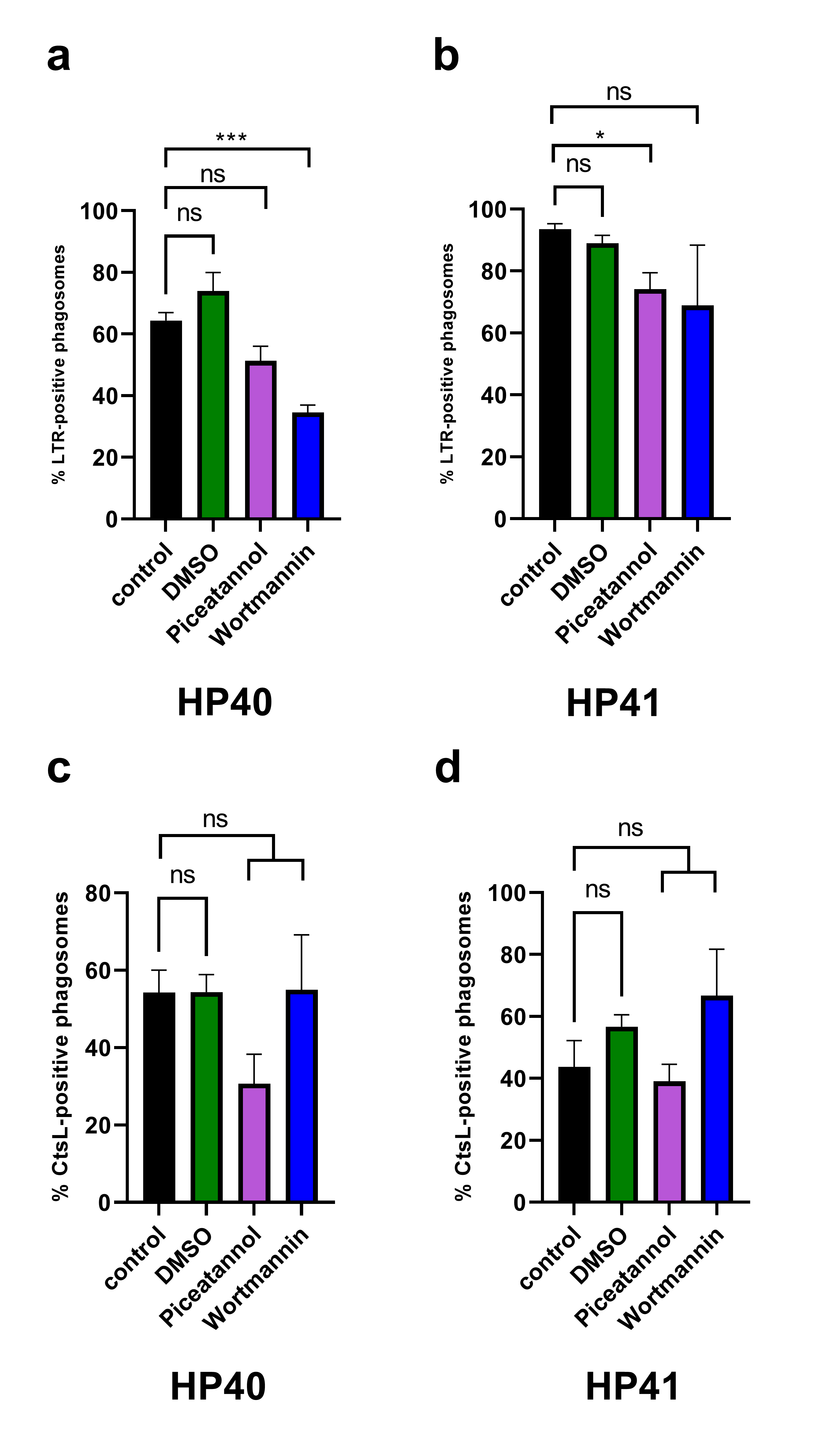

Supplement: Fig. S3 — Pharmacological inhibition of Syk or PI3K has variable effects on the maturation of P. bovis (HP40 and HP41) phagosomes. [file iai.00066-23-s0003.tif]

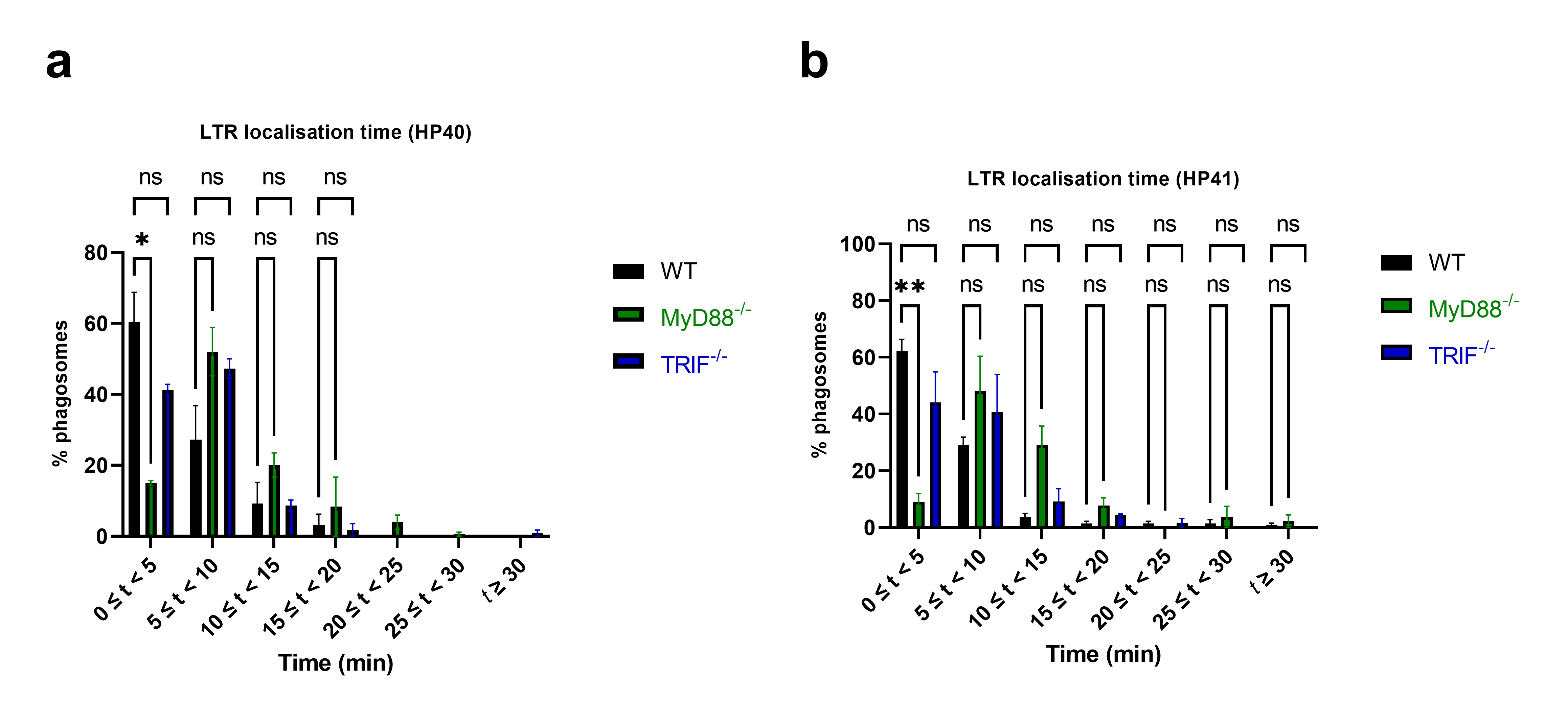

Supplement: Fig. S4 — Genetic ablation of MyD88 significantly impacts maturation of P. bovis (HP40 or HP41) phagosomes. [file iai.00066-23-s0004.tif]
