# Supplementary material for: Species- and strain-specific differences in the phagocytosis of Prototheca: insights from live-cell imaging
Source: Infect Immun. 2023 Aug 18;91(9):e00066-23. doi: 10.1128/iai.00066-23 (PMC10501220; doi:10.1128/iai.00066-23)
Supplement: Supplemental material legends and descriptions — Text-based description of supplemental figures, tables, and videos with legends. [file iai.00066-23-s0005.docx]

**Fig. S1. Uptake and phagosome maturation dynamics for *P. bovis* and *P. wickerhamii* phagosomes**. J774A.1 cells were seeded at 1 X 10^5^/well and incubated at 37^o^C and 5% CO_2_. The next day, cells were challenged with HP40, HP41, or HP50 at an MOI = 3 in the presence of LTR and imaged every 3 min for 6 h on a Zeiss Axio Observer Live microscope using a 20X objective. Data are expressed as the mean ± SEM of two independent experiments and show (**a**) the percentages of phagosomes requiring different periods of time for closure, (**b**) the percentages of phagosomes requiring different periods of time for LTR localisation, and (**c**) the percentages of actively phagocytosing macrophages that take up varying numbers of algal cells by the end of the imaging period. (**d**) Co-cultures were set up as described above and incubated for 6 h at 37^o^C and 5% CO_2_. Cells were then lysed in sterile water, diluted, plated out on *Prototheca* isolation medium (PIM) agar, and incubated at 25^o^C for 48 h. CFUs were then enumerated. Percentages shown in red beneath the x-axis describe % recovery from initial inocula. (**a – c**) Statistical significance was assessed by 2-way ANOVA followed by Šídák's multiple comparisons test where *p*<0.05 was considered significant. (**d**) Statistical significance was assessed by an unpaired *t-*test where *p* < 0.05 was considered significant. ns = not significant, **p*<0.05, ** *p* < 0.01.

**Fig. S2. Uptake and phagosome maturation dynamics for *P. bovis* phagocytosed by J774A.1 cells and WT iBMDMs**. J774A.1 cells and WT iBMDMs were seeded at 5 X 10^4^/well and incubated at 37^o^C and 5% CO_2_. The next day, cells were challenged with HP40 or HP41 at an MOI = 3 in the presence of LTR or CtsL and imaged every 30 sec for 1 h on a Nikon Eclipse Ti Live microscope using a 20X objective. Data are expressed as the mean ± SEM of three independent experiments and show (**a**) the percentages of phagosomes requiring different periods of time for closure, (**b**) the percentages of actively phagocytosing macrophages that take up varying numbers of algal cells by the end of the imaging period, (**c**) the percentages of phagosomes requiring different periods of time for LTR localisation, (**d**) the percentages of LTR-positive phagosomes overtime, (**e**) the percentages of phagosomes requiring different periods of time for CtsL localisation, and (**f**) the percentages of CtsL-positive phagosomes overtime. Statistical significance was assessed by 2-way ANOVA followed by Šídák's multiple comparisons test where *p*<0.05 was considered significant. ns = not significant.

**Fig. S3. Pharmacological inhibition of Syk or PI3K has variable effects on the maturation of *P. bovis* (HP40 and HP41) phagosomes.** WT iBMDMs were seeded at 5 X 10^4^/well and incubated at 37^o^C and 5% CO_2_. The next day, cells were treated for 1 h with DMSO (1mM), the Syk inhibitor Piceatannol at 75 μM, or the PI3K inhibitor Wortmannin at 20 μM in the presence of LTR or CtsL, and then challenged for 1 h with HP40 or HP41 at an MOI = 3. Cells were then fixed in 4% paraformaldehyde, washed, and imaged on a Nikon Eclipse Ti Live microscope using a 20X objective. Data are expressed as the mean ± SEM of three independent experiments and show (**a** and **b**) %LTR-positive phagosomes for HP40 and HP41, respectively, and (**c** and **d**) %CtsL-positive phagosomes for HP40 and HP41, respectively. Statistical significance was assessed by an unpaired t-test where *p* < 0.05 was considered significant. ns = not significant. * *p* < 0.05, *** *p* < 0.001.

**Fig. S4. Genetic ablation of MyD88 significantly impacts maturation of *P. bovis* (HP40 or HP41) phagosomes**. WT, MyD88^-/-^, or TRIF^-/-^ iBMDMs were seeded at 5 X 10^4^/well and incubated at 37^o^C and 5% CO_2_. The next day, cells were challenged with HP40 or HP41 at an MOI = 3 in the presence of LTR and imaged every 30 sec for 1 h on a Nikon Eclipse Ti Live microscope using a 20X objective. Data are expressed as the mean ± SEM of at least two independent experiments and show the percentage of (**a**) HP40 or (**b**) HP41 phagosomes requiring different periods of time for LTR localisation. Statistical significance was assessed by 2-way ANOVA followed by Dunnett’s multiple comparisons test where *p* < 0.05 was considered significant. ns = not significant. * *p* < 0.05, ***p*<0.01.

**Table S1. *P. wickerhamii* (HP50) phagosome closure time.**

**Table S2. *P. wickerhamii* (HP50) phagosome LTR localisation time.**

**S1 Vid. J774A.1 cells stained with LTR phagocytosing *P. bovis* (HP40).**

**S2 Vid. J774A.1 cells stained with LTR phagocytosing *P. bovis* (HP41).**

**S3 Vid. Human MDMs stained with LTR phagocytosing *P. bovis* (HP40)**

**S4 Vid. Human MDMs stained with LTR phagocytosing *P. wickerhamii* (HP50)**

**S5 Vid. WT iBMDMs stained with LTR phagocytosing *P. bovis* (HP40).**

**S6 Vid. WT iBMDMs stained with LTR phagocytosing *P. bovis* (HP41).**

**S7 Vid. J774A.1 cells stained with CtsL phagocytosing *P. bovis* (HP40).**

**S8 Vid. J774A.1 cells stained with CtsL phagocytosing *P. bovis* (HP41).**

**S9 Vid. WT iBMDMs stained with CtsL phagocytosing *P. bovis* (HP40).**

**S10 Vid. WT iBMDMs stained with CtsL phagocytosing *P. bovis* (HP41).**
