# Supplementary material for: Species- and strain-specific differences in the phagocytosis of Prototheca: insights from live-cell imaging
Source: Infect Immun. 2023 Aug 18;91(9):e00066-23. doi: 10.1128/iai.00066-23 (PMC10501220; doi:10.1128/iai.00066-23)
Supplement: Tables S1 and S2 — P. wickerhamii (HP50) phagosome closure time and LTR localization time. [file iai.00066-23-s0006.docx]

**Table 1. *P. wickerhamii* (HP50) phagosome closure time.**

| **Treatment** | **Total number of phagosomes analysed** | **Phagosome closure time (min)** |
| --- | --- | --- |
| Unopsonised | 0 | NA |
| Opsonised | 27 | 3.30 ± 0.41 |

**Table 2. *P. wickerhamii* (HP50) phagosome LTR localisation time.**

| **Treatment** | **Total number of phagosomes analysed** | **LTR localisation time (min)** |
| --- | --- | --- |
| Unopsonised | 0 | NA |
| Opsonised | 20 | 6.15 ± 2.01 |
